# Dr. Walter Vought

**Published:** 1893-11

**Authors:** 


AT A meeting of the New York Neurological Society, held October 3, 1893, the following resolutions were adopted :

*Resolved*, That it is with the deepest regret that this society has learned of the death of Dr. Walter Vought, one of its youngest and most promising members, and one who had already given evidence of the possession of exceptional knowledge and ability, not only in the department of neurology, but in the whole field of general medicine,

*Resolved*, That in the death of Dr. Vought, his colleagues lose a friend much esteemed for his excellence as a physician, and still more for his good-fellowship, loyalty, devotion to principle, and nobility of character.

*Resolved*, That this society in expressing its appreciation of the high qualities of heart and brain of its departed member, and its feeling of regret at his loss, desires in addition to extend its deep sympathy to his bereaved family.

FREDERICK PETERSON, M. D.,
JOHN W. BRANNAN, M. D.,
*Committee.*

